# Being watched: Effects of an audience on eye gaze and prosocial behaviour

**DOI:** 10.1016/j.actpsy.2019.02.002

**Published:** 2019-04

**Authors:** Roser Cañigueral, Antonia F. de C. Hamilton

**Affiliations:** UCL Institute of Cognitive Neuroscience, Alexandra House, 17 Queen Square, London WC1N 3AZ, UK

**Keywords:** Being watched, Audience effect, Reputation management, Prosocial behaviour, Eye gaze, Dual function of gaze

## Abstract

When someone is watching you, you may change your behaviour in various ways: this is called the ‘audience effect’. Social behaviours such as acting prosocially or changing gaze patterns may be used as signals of reputation and thus may be particularly prone to audience effects. The present paper aims to test the relationship between prosocial choices, gaze patterns and the feeling of being watched within a novel ecologically valid paradigm, where participants communicate with a video-clip of a confederate and believe she is (or is not) a live feed of a confederate who can see them back. Results show that when participants believe they are watched, they tend to make more prosocial choices and they gaze less to the confederate. We also find that the increase in prosocial behaviour when being watched correlates with social anxiety traits. Moreover, we show for the first time that prosocial choices influence subsequent gaze patterns of participants, although this is true for both live and pre-recorded interactions. Overall, these findings suggest that the opportunity to signal a good reputation to other people is a key modulator of prosocial decisions and eye gaze in live communicative contexts. They further indicate that gaze should be considered as an interactive and dynamic signal.

## Highlights

• The belief in being watched tends to increase prosocial choices of participants.
• The increase in prosocial behaviour correlates with higher social anxiety traits.
• Participants gaze less to the confederate when they believe she can see them.
• Less prosocial choices correlate with more gazes to confederate after the choice.
• The opportunity to signal good reputation modulates prosocial choices and eye gaze.

## 1 Introduction

We naturally care about how other people judge us, that is, our reputation. When our reputation is at stake, we change our behaviour in order to maintain it, because this makes us appear likeable to others (Emler, 1990; Tennie, Frith, & Frith, 2010). A subtle but recurrent ‘threat’ to our reputation is whether other people are watching us or not. The present paper explores how the belief in being watched modulates two behaviours that acquire a signalling function in the presence of an observer: prosocial decisions (Bradley, Lawrence, & Ferguson, 2018; Izuma, Matsumoto, Camerer, & Adolphs, 2011) and eye gaze (Gobel, Kim, & Richardson, 2015; Laidlaw, Foulsham, Kuhn, & Kingstone, 2011). We study these changes in a conversation context, using a novel well-controlled experimental paradigm. For the first time we also examine the relationship between gaze of participants and their prosocial choices, and propose that this relationship can help identifying which social cognitive processes modulate gaze behaviour in live versus pre-recorded interactions. In the following, we briefly review studies of how people respond when being watched in a variety of contexts.

### 1.1 Reputation management and being watched

Theories about how people change their behaviour in the presence of other people were first introduced by Triplett in 1898, when he discovered that cyclists were faster when competing against each other than against a clock (Triplett, 1898). He stated that the ‘bodily presence of another’ caused a change in the behaviour of participants, making them more competitive when racing. It is important here to distinguish between ‘social facilitation’ (Zajonc, 1965), which is an enhancement of performance in the presence of any conspecific (who may or may not be looking), and the ‘audience effect’, which is a change in behaviour specifically caused by the belief that someone else is watching me. Here we focus on the latter.

An increasing number of studies suggest that the audience effect can best be understood in terms of reputation management (Emler, 1990; Resnick, Zeckhauser, Swanson, & Lockwood, 2006; Tennie et al., 2010). Reputation is a social construct that emerges from how we think others see us, and is changeable over time depending on our actions (Cage, 2015; Izuma, 2012). For instance, acting for the benefit of other people and conforming to social norms are two examples of how individuals can signal their good reputation to gain approval of others. The maintenance or management of reputation requires individuals to infer what others think of them, care about how they are seen, and have the desire to be viewed positively (Cage, 2015; Izuma, 2012). This means that reputation management requires both mentalizing and social motivation (Cage, 2015; Izuma, 2012; Izuma, Saito, & Sadato, 2010; Tennie et al., 2010). This is supported by neuroimaging studies showing that brain regions involved in these two cognitive processes are activated during different phases of reputation management. For instance, the medial prefrontal cortex (a neural correlate for mentalizing; Frith & Frith, 2006) is activated when processing one's reputation in the eyes of other people (Izuma, 2012; Izuma et al., 2010). Moreover, a region involved in motivation and reward processing, the ventral striatum, is engaged when participants anticipate positive reputation after presenting themselves in front of others (Izuma, 2012; Izuma et al., 2010; Izuma, Saito, & Sadato, 2009).

When people are observed by others, one way to signal their reputation is by behaving in a more prosocial fashion (Bradley et al., 2018; Smith & Bird, 2000). Several real-life studies have shown that the possibility of gaining reputation in front of others is a key factor to increase prosocial behaviour (e.g. Bereczkei, Birkas, & Kerekes, 2007; Raihani & Smith, 2015; Soetevent, 2005). Lab-based studies, which allow for more experimental control, also show similar results. For instance, Satow (1975) used a single-trial task and found that in the presence of an experimenter participants donate more money to a research fund than in its absence. Other studies have used economic games, which facilitate reputation building between subjects in the game by having more trial repetitions than single-trial tasks (Bradley et al., 2018; Pfeiffer & Nowak, 2006). Using the Public Goods game, Filiz-Ozbay and Ozbay (2014) showed that being watched by another participant increases the amount of effort exerted to contribute to public, but not private, goods. In another study Izuma and colleagues used the Dictator game (Izuma et al., 2011): on each trial participants were given a specific amount of money and had to decide whether to give some of this money to someone else (e.g. charity; prosocial behaviour) or keep it all for themselves (non-prosocial behaviour). They found that participants donated money more often while monitored by a confederate than when alone in a room, which can be interpreted as reputation management. Cage and colleagues (Cage, Pellicano, Shah, & Bird, 2013) replicated this finding and also found that, when the recipient was an individual (not a charity) who could later reciprocate to the participant, the number of donations was higher in the presence than in the absence of an observer. These studies are clear examples of participants manipulating the information they signal to other people in order to maintain good reputation.

These studies have two main limitations. On the one hand, the control and test conditions are not optimally matched to strictly isolate effects of the belief in being watched: they compare a control condition where the participant is alone in the room, versus a test condition where an observer is present in the room or in a video-feed (see Izuma et al., 2010, Izuma et al., 2009 for examples of studies with a video-feed). Instead, control and test conditions that are both social would be more suitable to test true audience effects. The present paper uses more closely matched experimental conditions that vary only in the belief in being seen, to understand how a belief manipulation alone (without any changes in the presence of the confederate) affects reputation management. On the other hand, although prosocial behaviour has been traditionally measured by economic games, such as the Public Goods or Dictator games, concerns have been raised about their external validity (Galizzi & Navarro-Martinez, 2017; Winking & Mizer, 2013). Thus, in the present study we compare how the belief in being watched modulates prosocial behaviour in the Dictator game and in a novel task where participants disclose their prosocial tendencies in everyday life situations.

### 1.2 Gaze behaviour and being watched

Our eyes have a dual function in social interactions: they gather information from the world, but also send signals to other people (Gobel et al., 2015; Risko, Richardson, & Kingstone, 2016). For instance, direct gaze signals a desire to communicate (Ho, Foulsham, & Kingstone, 2015; Kendon, 1967), it monitors facial displays of the other person to ensure mutual understanding (Kleinke, 1986), it expresses affiliation or (dis)agreement (Kendrick & Holler, 2017), attractiveness (Georgescu et al., 2013), and threat or dominance (Emery, 2000; Gobel et al., 2015). Conversely, averted gaze has been linked to preference for no interaction (Foulsham, Walker, & Kingstone, 2011), conformity with social or cultural norms (Gobel, Chen, & Richardson, 2017; Gobel et al., 2015; Laidlaw et al., 2011; also known as ‘civil inattention’, Goffman, 1963), and fear or submissive behaviour (Emery, 2000; Gobel et al., 2015). The variety of social meanings that our eyes can convey makes our gaze a powerful tool for social interactions.

Although the dual function model of eye gaze was first introduced in the 70s (Argyle & Cook, 1976), many studies have ignored it. In traditional experimental settings, participants see pictures or videos of a person while their gaze or other actions are recorded (see Risko, Laidlaw, Freeth, Foulsham, & Kingstone, 2012 for a review), but they are fully aware that the pictures or videos cannot see back. Thus, participants are not signalling anything to the person in the stimulus because it makes no sense to communicate with a picture unable to perceive them. These traditional approaches allow good experimental control but are not interactive (Gobel et al., 2015; Schilbach et al., 2013), and it is increasingly recognised that understanding the cognitive mechanisms of social behaviour will require more than just one-way picture stimuli.

A few recent studies have examined how people's gaze behaviour changes when they believe they are being watched, that is, when gaze acquires a signalling function. For instance, Laidlaw et al. (2011) measured the looking behaviour of participants with eye-tracking as they were sitting in a waiting room, either in a presence of a confederate or in the presence of a video-clip of the same confederate. It was found that participants tended to look at the confederate in the video-clip, but seldom looked at the live confederate. In another study, Gobel and colleagues (Gobel et al., 2015) used eye-tracking to explore how participants changed gaze patterns when they believed they would later be viewed by another person. Participants watched video-clips of high and low rank people while their face was recorded. Results showed that, if participants believed the person in the video would later see the recordings, they made more eye contact with the low rank model, and less with the high rank model. In these studies, the authors suggest that averted gaze in live (versus pre-recorded) settings signals the activation of previously acquired social norms, by which it is not polite to stare at someone (Gobel et al., 2017). The effect of these social norms translates into active gaze disengagement because participants do not want to appear as either someone impolite or as an interaction partner to the stranger (Foulsham et al., 2011).

There is a main limitation to these previous studies: participants believe they are interacting with a stranger with whom they are not supposed to talk to, that is, there is no communicative exchange between them. These results may not generalise to all social contexts. For instance, it has recently been shown that it is the potential for social interaction, rather than online social presence, which modulates eye gaze in video-conference contexts (Gregory & Antolin, 2018). Mansour and Kuhn (2019) have also shown that when participants are required to actively engage with the confederate, they direct more gaze to the eyes of the confederate in the live video-call than in the pre-recorded video-call. Thus, communicative (e.g. conversation) and non-communicative environments may engage a series of cognitive processes that modulate differently the amount of gaze directed to a live person. In the present study, we test if gaze signalling patterns change between a live and pre-recorded setting in the context of a question-answer task, where it is clear that participant and confederate should communicate.

### 1.3 Relationship between prosocial and gaze behaviour

In communicative situations we send information through eye gaze, but also through speech, facial expressions and gestures. To further understand the meaning of gaze patterns, it is useful to consider gaze in relation to other events in the communicative exchange: this can help identifying which cognitive mechanisms modulate eye gaze in live interactions. Previous studies on eye gaze have found that eye contact elicits more prosocial behaviour (Bull & Gibson-Robinson, 1981) and that we engage in mutual gaze to seek approval from others (Efran, 1968; Efran & Broughton, 1966). However, we are not aware of previous studies examining temporal relationships between gaze patterns and prosocial behaviour. Thus, a core question of the present paper is to see if and how these behaviours are related. We can draw out at least two plausible hypotheses.

First, we can consider how gaze patterns before a prosocial decision relate to what decision is made. For example, if two people share mutual gaze, this may increase their prosocial behaviour (see Bull & Gibson-Robinson, 1981 for an example). Similarly, gaze to another person can be an indicator of how much you are interested in that person or care about them, which might predict later prosocial responses to that person. In this case, a relationship between gaze patterns before making a choice and a prosocial choice itself would indicate that social attention influences prosocial choices (social attention hypothesis). This could occur regardless of whether the participant is interacting with a video or another person.

Second, we can consider how making a prosocial or antisocial decision changes gaze patterns after this decision. For example, after making a donation to a charity someone may look to others to receive their approval or to seek more information about what they think (Efran, 1968; Efran & Broughton, 1966; Kleinke, 1986). Building on this idea, we suggest that if there is a relationship where choices predict later gaze patterns, this might indicate that participants are engaged in a process of reputation management (reputation management hypothesis). However, this should only occur if people believe they are engaged in a live interaction with a real person.

Thus, the relationship between gaze patterns and prosocial choices can help us understand some of the underlying cognitive processes which drive these behaviours, and show if either social attention or reputation management are important in these contexts.

### 1.4 The present study

The present study aims to gain a better understanding of how the belief in being watched modulates prosocial and gaze behaviours as signals to maintain a good reputation. Our specific aims are the following. First, to compare whether two different types of prosocial behaviour that can signal good reputation - monetary donations and disclosure of prosocial tendencies - show similar changes between a live and pre-recorded interaction. Economic games have been recently reported to have poor external validity (Galizzi & Navarro-Martinez, 2017; Winking & Mizer, 2013), so it is helpful to know whether changes in monetary donations and changes in disclosure of prosocial tendencies are consistent. Second, to examine the signalling function of eye gaze (between a live and pre-recorded interaction) when participant and confederate are in a communicative situation. This will clarify whether results from previous studies using non-communicative contexts (Gobel et al., 2015; Laidlaw et al., 2011) generalise to other social contexts. Finally, we aim to study for the first time the relationship between prosocial behaviour and eye gaze. This can help us understand which cognitive processes - social attention or reputation management - drive these behaviours.

**Fig. 1:**
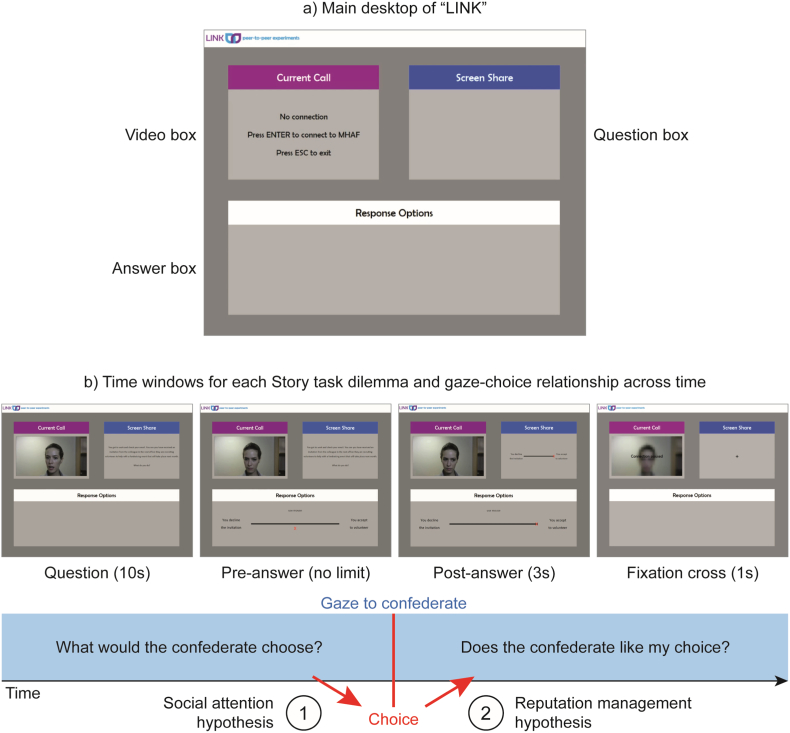
a) Main desktop of the fake video-conference interface “LINK”. b) Screenshots of the time windows for each dilemma/trial of the Story task, and model describing potential relationships between gaze and prosocial choices across the different time windows.

To do this, we designed a deceptive video-conference interface that participants would use to complete the study. This novel experimental paradigm allows for well-matched control and test conditions but at the same time preserves enough ecological validity (see Mansour & Kuhn, 2019 for a recent paper using a similar paradigm), which ensures that changes in behaviour are true audience effects. The main desktop of the interface showed three different boxes: the Video box, where the video-feed was presented, the Question box, where the questions appeared, and the Answer box, where the options for the answer were shown (see Fig. 1a). In our deceptive manipulation we used the same video-clips of two confederates across two settings: one where participants believed the video-feed was real (online setting; ON), and one where they were told the videos were pre-recorded (offline setting; OFF). This ensured high ecological validity for the ON setting and, at the same time, the use of well-matched stimuli across ON and OFF settings. Participants believed the two confederates were students volunteering in a charity.

In our within-subject design, participants completed two tasks measuring prosocial behaviour. Participants played the Dictator game used by Izuma et al. (2011), where we measured the frequency of accepted donations (Offer task). Although prosocial behaviour has been traditionally measured by economic games, such as the Dictator game, concerns have been raised about their external validity (Galizzi & Navarro-Martinez, 2017). For this reason, we also used a novel Story task inspired by Izuma et al. (2010), where participants disclose their prosocial tendencies in everyday life situations. During the task, we ensured a communicative environment by 1) having videos where the confederate read the questions to the participant, and 2) telling participants to say their choice aloud before entering it in the computer. Based on previous evidence (Cage et al., 2013; Izuma et al., 2011), we hypothesized that the belief in being watched would increase prosocial behaviour of participants across both tasks, because it signals good reputation to the observer.

During the tasks, participants' eye gaze was recorded with eye-tracking, and we measured the looking time to the three boxes on the screen – the Video box, the Question box, and the Answer box. We can contrast two possible hypotheses for gaze behaviour. If in our communicative context participants need to gain or signal useful information from/to the live confederate, then they might look more to the Video box under the ON setting compared to OFF setting. However, if participants still conform to a social norm of avoiding staring, we may replicate the results of Gobel et al. (2015) and Laidlaw et al. (2011), and find more gaze to the Video box under the OFF setting.

A core question in this study concerns the relationship between prosocial choices and gaze directed at the confederate (Video box) on a trial-by-trial basis. The presence and direction of this relationship across different time windows can help identifying which social cognitive processes modulate gaze behaviour (see Fig. 1b). As introduced earlier, we will test if gaze before the choice predicts the later choice behaviour (social attention hypothesis), and if the choice predicts gaze behaviour during the post-answer phase (reputation management hypothesis). Importantly, we expect that the social attention hypothesis will be true for both settings, while the reputation management hypothesis will only happen in the ON setting.

After the tasks, participants filled a questionnaire about their perception of the confederates in each setting, and a questionnaire measuring their social anxiety traits. People with social anxiety fear or perceive negatively other people, and they show increased concern to gain social approval (Cremers & Roelofs, 2016; Morrison & Heimberg, 2013). A meta-analysis by Uziel (2007) shows that negative personality traits (e.g. low self-esteem, neuroticism or introversion, which are associated with social anxiety) are strong predictors of how social presence will affect individual performance. In line with this, Satow (1975) found that, when answers were public, people in high need for social approval (i.e. those who score high in the Social Desirability Scale; Crowne & Marlowe, 1960) donated more money than people in low need for social approval. This indicates that people with social anxiety traits might be more susceptible to audience effects. Here, we perform an exploratory analysis of the relationship between social anxiety traits and audience effects.

## 2 Materials and methods

### 2.1 Participants

We aimed for a sample of 32 participants (8 for each condition). Overall, a group of 43 adults (25 females, 18 males; mean age: 23.95 ± 3.59) were recruited because, as we were testing, 9 participants did not believe the deceptive manipulation for the online setting, and 2 participants had poor-quality eye-tracking data. Thus, the final valid sample consisted of a group of 32 adults (20 females, 12 males; mean age: 23.41 ± 3.55). All participants gave written informed consent before doing the experiment and were compensated £8 for their time and travel expenses; they were aware that they could receive a bonus of maximum £4 depending on their performance during the Offer task (see Section 2.5 for details on the Offer task bonus). The study was granted ethical approval by the local Research Ethics Committee, and was in accordance with the Declaration of Helsinki.

### 2.2 Cover story

In order to manipulate the beliefs of participants in an efficient and credible way, participants were told that we were investigating social attention during charitable behaviour, and that they would complete a task with two student volunteers working in a charity (confederates). Participants were given an information sheet about the aims and work of the charity. Although the name of the charity was not real (Mental Health Awareness Foundation), the description was very similar to that of the real charity Mental Health Foundation and money collected during the task was donated to the latter.

Participants were told that we would connect online with the two confederates at the charity using “an interface similar to Skype but for experimental research” that we called “LINK: peer-to-peer experiments”. The experimenter pretended to launch LINK through MATLAB. However, the screens shown during the task were designed with MATLAB (R2016b, MathWorks) and Cogent Graphics in a way that tried to escape from the typical experimental layout. The LINK main desktop would show a banner on the top with the LINK logo, a box called Current Call (where the video call would appear; Video box in the analyses), a Screen Share box (both the participant and the confederate were supposed to see this box; the questions and chosen answers were displayed here; Question box in the analyses), and the Response Options box (where the participant could see the option to answer the question; Answer box in the analyses) (see Fig. 1a). Participants were also told in the beginning that, in case the students in the charity (confederates) were not available, a set of videos recorded during the piloting of the study would be used instead.

### 2.3 Counterbalancing conditions

There were four different conditions, in which we counterbalanced the order of the settings (online = ON, offline = OFF), the confederate linked to each setting and session (confederate 1, confederate 2), and the story linked to each setting and session (story 1, story 2) (see S1 for a table with all counterbalancing conditions). Each participant was allocated to one condition: they completed the Story and Offer tasks twice, one for each setting.

### 2.4 Story task

In order to test how the audience effect changes reputation management, we designed a task inspired by Izuma et al. (2010), where participants have to disclose their tendencies relative to social norms. We created a set of 2 stories that depicted real day-to-day situations emulating a moral dilemma. These moral dilemmas were part of a larger pool of dilemmas that we created and piloted through an online form on 23 adults. In each story, there were 5 different dilemmas (i.e. 5 trials) with two options: one option was prosocial but had a temporal or monetary cost (e.g. volunteer for an afternoon, give money to a homeless person), whereas the other option was non-prosocial and had no cost (see S2 for full stories). Both stories had an additional neutral trial where both options were non-prosocial.

For each trial, the confederate in the video read a statement describing the dilemma and asked participants ‘What do you do?’. Participants could also read the statement on the Screen Share. The two possible answers were displayed on each end of a continuous scale in the Response Options box, and participants indicated with the mouse how likely they were to do one or the other option (halfway the line was a neutral answer). Participants were instructed to say their choice aloud to the confederate before clicking the mouse, in order to create a communicative environment. The choice was displayed on the Screen Share for 3 s, and the confederate in the video stayed in silence as if she was checking the choice. In between trials a fixation cross was displayed on the Screen Share for 1 s, and a blurred frame of the video-clip plus the message ‘Connection paused' were displayed on the Current Call box (see Fig. 1b for screenshots of each time window and S3 for a sample trial).

### 2.5 Offer task

**Fig. 2:**
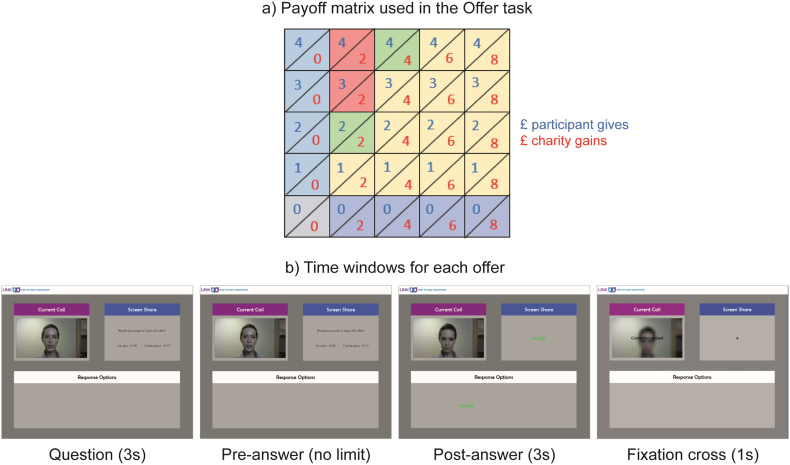
a) Payoff matrix. b) Screenshots of the time windows for each offer/trial of the Offer task.

As a second measure of the audience effect, we used a variation of the Dictator game previously used by Izuma et al. (2011) and Cage et al. (2013). We used a modified version of the payoff matrix used by Cage et al. (2013), in which we reduced the amounts at play to adapt them to our participation fee (see Fig. 2a). Each cell in the payoff matrix corresponds to one trial, which was tested once for each setting (ON, OFF); within each setting, the 25 trials were randomized. To avoid participants memorizing their choices, we applied a jittering on the amounts of money by adding a random number from a normal distribution N(0,0.2). If the original amount was 0, no jittering was applied; if the amounts the participant would give and the charity would gain were equal, the jittering was the same for both amounts. The trials in which the participant would give £0 and the charity would gain £0 were removed from the analyses since the choices would be random.

For each offer, the confederate in the video asked to the participant ‘would you accept or reject this offer?’, and both the question and the monetary offer were displayed on the Screen Share. The two possible answers (‘accept’ and ‘reject’) were displayed on the Response Options box, and the side where they appeared was counterbalanced across trials. To select an option, participants had to press a blue key (‘D’ or ‘K’) that matched the position of the chosen option. Participants were instructed to say the answer aloud to the confederate before pressing the key. After the key press, the answer was displayed on the Screen Share for 3 s, during which the confederate in the video stayed in silence as if she was looking at the answer. In between trials, a fixation cross was displayed on the Screen Share for 1 s, while a blurred frame of the video-clip plus the words ‘Connection paused’ were displayed on the Current Call box (see Fig. 2b for screenshots of each time window).

Importantly, in the beginning participants were told that, on top of the fixed payment of £8, they would receive a bonus of maximum £4 depending on their choices in the Offer task. They were told that in the end of the experiment a random trial would be selected: if in that trial participants had accepted the offer, they would give that amount to the charity and keep the rest; conversely, if they had rejected the offer, they would keep the full £4 bonus.

### 2.6 Stimuli: video-clips

We recorded 3 sets of video-clips for each of the two confederates: Alice and Sophie. During the filming session, the confederate went through the two tasks and was recorded with a webcam on top of a monitor, in order to simulate an online connection. The first set of video-clips was composed of 2 different videos where the confederate was pretending to have a conversation with someone else, although only her part of the dialogue was recorded: in the first conversation she was greeting the participant and experimenter, testing that the Screen Share worked, and receiving the instructions for the Story and Offer tasks; in the second conversation she said goodbye to the participant and experimenter. The second set of video-clips was composed of 6 short videos for the Story task (one for each trial): for each video-clip, the confederate would first look at the screen and read a statement, then look at the camera and ask a question, and finally look at her screen again for 10 s. The third set of video-clips was composed of 25 short videos for the Offer task (one for each trial). For each video clip, the confederate would first look at her screen for 1–2 s, then look at the camera and ask the question, and finally look back to her screen for 10 s.

### 2.7 General procedure: deceptive video-conference paradigm

**Fig. 3:**
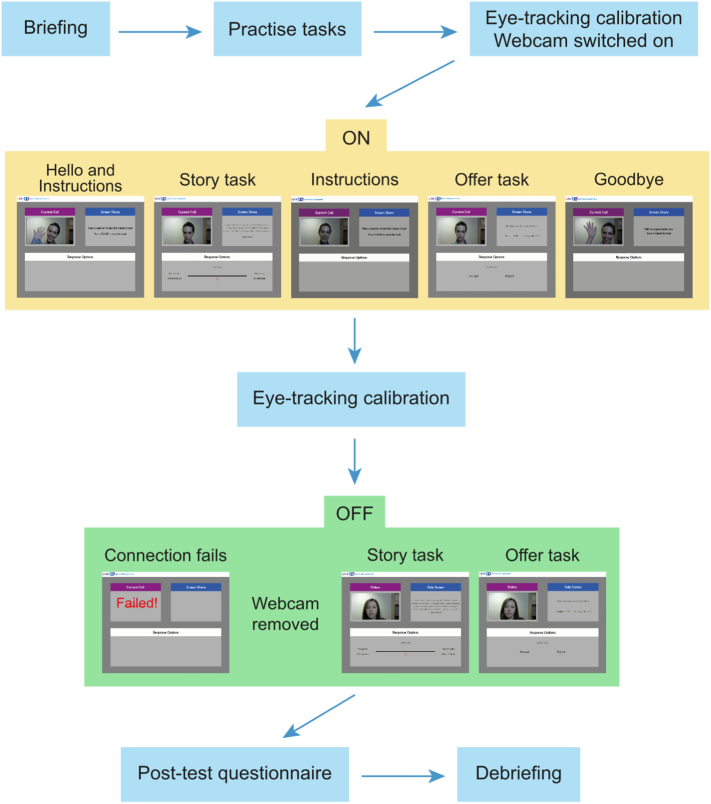
Overview of the procedure for each participant.

As an example, below we present the procedure for conditions 1 and 2, where participants complete the tasks under the ON setting and then under the OFF setting (Fig. 3).

Once the participant had read the information about the charity and practised the two tasks without video-clips, the eye-tracker was calibrated. The experimenter also pretended to check the webcam was working by launching the ‘Webcam video’ on Movie Maker and leaving it open, so the green light on the webcam would indicate it was switched on. The experimenter loaded LINK and explained to the participant the meaning of the boxes on the LINK main desktop. Following the instructions on the Current Call box, the experimenter would then connect to the charity.

For the ON setting, the connection was successful and the video of the confederate (Alice) was played. Although the video was pre-recorded, the experimenter pretended to have a conversation with Alice and she had previously rehearsed its timing to ensure credibility. During the conversation, the experimenter introduced Alice to the participant and pretended to run a test with Alice to check the Screen Share was working, thereby enhancing the belief that Alice was real and could see the information shown on the Screen Share. The experimenter then gave some instructions for the Story task to both Alice and the participant, explicitly telling Alice ‘don't make any facial expression or say anything that could influence the participant's choices’, so that the participant would not suspect of Alice being too unresponsive (see S4 for the full conversation). The experimenter left the testing room and waited outside until the participant announced the task was completed. Then the experimenter loaded the Offer task and gave instructions to both Alice and the participant, and left the testing room again until the participant announced the task was completed. Then, a short video of Alice saying goodbye was played. In between settings, the eye-tracker was calibrated again to make sure the data was recorded properly for the OFF setting.

For the OFF setting, the connection would fail, automatically try to connect again, and fail again. Three options were displayed on the Current Call box: try the connection again, use the offline mode with stored videos, or exit. During this time, the experimenter pretended to get concerned about the connection and to send a text to the second confederate (Sophie). Shortly after, she pretended that Sophie had answered back saying that she was in a meeting that was taking longer than expected. At this point the experimenter told participants to use the pre-recorded videos: she would remove the webcam and load the offline mode of LINK. The LINK layout would change slightly: now the Current Call box was called Videos, and the Shared Screen was called Side Screen. Participants completed the tasks after receiving the corresponding instructions.

### 2.8 Post-test questionnaire and debriefing

After completing the two tasks under the two settings, all participants completed a post-test questionnaire that had 3 sections. In the first section, participants had to indicate on a scale from 0 (disagree) to 8 (agree) to what extent they agreed with some statements. These statements were related to their perception of the two models (e.g. ‘I liked Alice very much’) and the interaction with them (e.g. ‘I think the interaction with Alice was very natural’), and their perception of the relevance of the charity and charitable behaviour in their life (e.g. ‘I think it is very important to donate money to a charity’). In the second section, participants were asked some questions to check they did not realise the real purpose of the experiment and to know about their strategies to give an answer. Finally, in the third section participants completed the Liebowitz Social Anxiety Scale (LSAS; Liebowitz, 1987). It consists of 24 questions assessing social anxiety and phobia across different real life situations. The overall score can range from 0 (low social anxiety) to 144 (high social anxiety), with scores over 65 reflecting marked/severe social phobia. See S5 for the full post-test questionnaire.

After participants completed the post-test questionnaires, the experimenter ran the code to select the random trial that would determine how much participants kept from the £4 bonus. If participants were meant to give part of the bonus to the charity, they would place the corresponding amount in a collection box. Once the data collection was completed, the experimenter added up all the monetary amounts that participants had given and made a donation to the Mental Health Foundation. Finally, the experimenter asked whether they noticed that the confederate in the ON setting was a pre-recorded video, and subsequently debriefed participants about the manipulation, the real purpose of the experiment and the real name of the charity. The overall duration of the experiment was around 40 min.

### 2.9 Eye-tracking

An Eye Tribe ET1000 eye-tracker (IT University of Copenhagen, Denmark) was positioned at the base of a 19″ monitor. Participants sat approximately 50 cm from the screen, and placed their head on a homemade chin rest fixed on the table. They went through a 9-point calibration routine that took between 1 and 2 min; they completed the calibration twice, once before each setting was loaded. The eye-tracker recorded the eye movements of both eyes at a rate of 30 Hz.

Three time windows and 3 regions of interest (ROIs) were defined. The 3 time windows corresponded to 1) the period of time where the confederate asked the question (‘question’; around 10 s), 2) the period of time before clicking the mouse, where participants were thinking about the answer and saying it aloud (‘pre-answer’; unlimited) and 3) the period of time after participants clicked the mouse, during which the answer was displayed on the Screen Share (‘post-answer’; 3 s). The ROIs corresponded to 1) the Video box, 2) the Question box and 3) the Answer box (see Fig. 1a). To measure eye gaze, we computed the proportion of looking time, which corresponds to the amount of time that participants spent looking at each ROI (video box, question box and answer box) relative to the total duration of each time window (question, pre-answer, post-answer).

### 2.10 Data analyses

To check that the deceptive manipulation changed how the confederate was perceived by the participant, two-tailed paired *t*-tests between ON and OFF setting were computed for each of the traits rated in the post-test questionnaire: likeability, naturalness and reciprocity.

For prosocial behaviour, we compared choices under the ON setting to those under the OFF setting, taking also into account the order in which the two settings appeared. For the Story task, the prosocial option was matched to 1 and the non-prosocial option to 0, and we measured the percentage of prosociality of the choices. For the Offer task, the number of trials in which participants accepted to donate money to the charity was computed (range: from 0 to 24 trials). A 2-way repeated measures ANOVA with Setting (ON and OFF) as within-subject factor, Order of setting (first or second) as between-subject factor, and dependent variable Choice was performed for each task. Post-hoc pairwise comparisons using Bonferroni's adjustment were also computed. Moreover, Pearson correlations were computed to assess the relationship between prosocial behaviour and social anxiety scores: we tested whether a greater difference in prosocial choices between ON and OFF settings correlated with higher social anxiety traits.

For the eye-tracking measures, we tested the effect of the setting (ON, OFF) on the proportion of looking time to the Video box, Question box and Answer box in the three time windows (question, pre-answer, post-answer). Data for the three regions is not independent because participants can only look at one place at a time. Therefore, we analysed gaze to the three regions separately, using a two-way repeated measures ANOVA with Setting and Time window as within-subject factors for each task. Where sphericity could not be assumed, corrected *p*-values using the Huynh-Feldt estimate were used. Post-hoc pairwise comparisons using Bonferroni's adjustment were also computed. Here we did not test correlations with social anxiety traits, because they would be underpowered to correct for multiple comparisons when all possible combinations between time windows and boxes on the screen were taken into account.

A critical question concerns the relationship between gaze and prosocial behaviour on a trial-by-trial basis. We used different models to test our two hypotheses on this relationship (social attention hypothesis and reputation management hypothesis). First, we tested whether choice was predicted by the belief in being seen and gaze behaviour prior to giving an answer. We fitted a mixed ANOVA with Setting and Gaze (% looking time to Video box during question phase) as independent variables, Participant as random factor, and Choice as dependent variable. For the Story task we included 320 trials (32 participants, 2 settings, 5 social trials), and for the Offer task we included 1536 trials (32 participants, 2 settings, 24 offers). Second, we tested whether gaze behaviour after giving an answer was predicted by choice and belief in being seen: we fitted a mixed ANOVA with Setting and Choice as independent variables, Participant as random factor, and Gaze (% looking time to Video box during post-answer phase) as dependent variable.

Since data was not normally distributed for all measures, we performed a bootstrap analysis with 10,000 permutation tests for each of the analyses, and examined the probability that the results could have arisen by chance, given the distribution of our existing data. The pattern of results for the bootstrap analysis (i.e. results above or below *p* < .05) was identical to the classical ANOVA analyses, so we report only the classic ANOVAs.

## 3 Results

### 3.1 Manipulation check: post-test questionnaire ratings

**Fig. 4:**
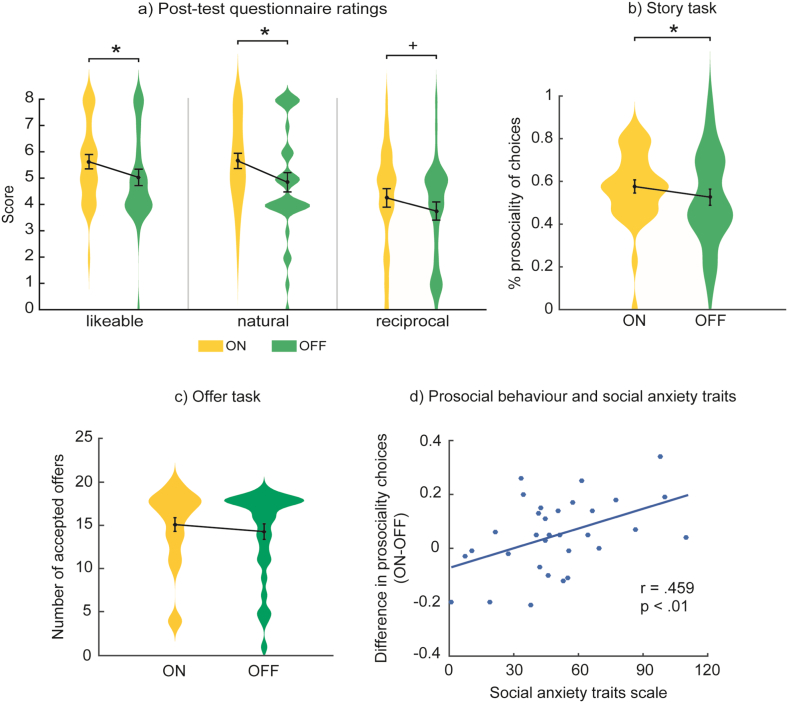
a) Post-test questionnaire ratings about the confederates: mean (filled circle), SE (error bars), and frequency of values (width of distribution). b) Percentage of prosociality of choices in Story task. c) Number of accepted offers in the Offer task. d) Correlation between prosocial behaviour and social anxiety traits in Story task. Asterisks signify difference at *p* < .05 (*), *p* < .01 (**) and *p* < .001 (***).

In the post-test questionnaire, participants rated the ON and OFF confederate on three traits: likeability, naturalness and reciprocity. Two-tailed paired *t*-tests between ON and OFF setting were computed for each trait. Results showed that under the ON setting the confederate was perceived as significantly more likeable, *t*(31) = 2.31, *p* < .05, d_z_ = 0.408, and natural, *t*(31) = 2.14, *p* < .05, d_z_ = 0.378, and tended to be perceived as more reciprocal *t*(31) = 1.72, *p* = .096, d_z_ = 0.304 (Fig. 4a). See Table 1 for descriptives (mean and SD) on post-test questionnaire ratings.

**Table 1:** Descriptives for post-test questionnaire ratings.

Rating	Setting	*M*	*SD*
Likeable	ON	5.62	1.54
	OFF	5.03	1.77
Natural	ON	5.66	1.64
	OFF	4.84	2.08
Reciprocal	ON	4.25	2.00
	OFF	3.75	1.95

### 3.2 Prosocial measures

To analyse prosocial measures, we fitted a 2-way repeated measures ANOVA for each task, with Setting (ON and OFF) as within-subject factor and Order of setting (first or second) as between-subject factor.

For the Story task, results showed a marginally significant effect of Setting on prosocial choices, *F*(1,30) = 4.16, *p* = .05, n_p_^2^ = 0.122 (Fig. 4b): choices were more prosocial under the ON setting (*M* = 0.576, *SD* = 0.174) than under the OFF setting (*M* = 0.526, *SD* = 0.215). There was no main effect of Order nor interaction between Setting and Order.

For the Offer task, there was a tendency to accept more offers under the ON setting (*M* = 15.1, *SD* = 4.49) than OFF setting (*M* = 14.3, *SD* = 5.07), *F*(1,30) = 3.43, *p* = .074, n_p_^2^ = 0.103 (Fig. 4c). There was no main effect of Order, but we found a tendency for an interaction between Setting and Order, *F*(1,30) = 2.92, *p* = .098, n_p_^2^ = 0.089: participants who performed the task first under the ON setting and then under the OFF setting showed no change in prosocial behaviour, whereas in the reversed order prosocial behaviour was lower in the OFF than in the ON setting.

Regarding social anxiety scores, we found a significant positive correlation between the change in prosocial behaviour (ON – OFF) and social anxiety traits for the Story task, *r* = 0.459, *p* < .01: the more participants changed their behaviour from OFF to ON setting, the more anxiety traits they had (Fig. 4d). There was no significant correlation between social anxiety traits and change in prosocial behaviour for the Offer task, *r* = 0.225, *p* > .05.

### 3.3 Eye gaze: story task

For eye gaze, we fitted a two-way repeated measures ANOVA for each box (Video, Question, Answer), with Setting (ON and OFF) and Time window (question, pre-answer, post-answer) as within-subject factors. See Table 2 for descriptives (mean and SD) on the proportion of looking time to each box and time window. Only significant main effects and interactions are reported in the text; full results and post-hoc tests are given in Supplementary materials (Table S6.1).

**Table 2:** Descriptives for the proportion of looking time to each box (Story task).

Setting	Time window	Video box	Question box	Answer box
ON	question	*M = 0*.094 *SD = 0*.093	*M = 0*.774 *SD = 0*.068	*M = 0*.045 *SD = 0*.032
	pre-answer	*M = 0*.010 *SD = 0*.015	*M = 0*.073 *SD = 0*.068	*M = 0*.861 *SD = 0*.096
	post-answer	*M = 0*.135 *SD = 0*.148	*M = 0*.447 *SD = 0*.153	*M = 0*.306 *SD = 0*.161
OFF	question	*M = 0*.148 *SD = 0*.082	*M = 0*.713 *SD = 0*.104	*M = 0*.037 *SD = 0*.029
	pre-answer	*M = 0*.016 *SD = 0*.023	*M = 0*.097 *SD = 0*.077	*M = 0*.816 *SD = 0*.119
	post-answer	*M = 0*.135 *SD = 0*.117	*M = 0*.472 *SD = 0*.166	*M = 0*.274 *SD = 0*.149

**Fig. 5:**
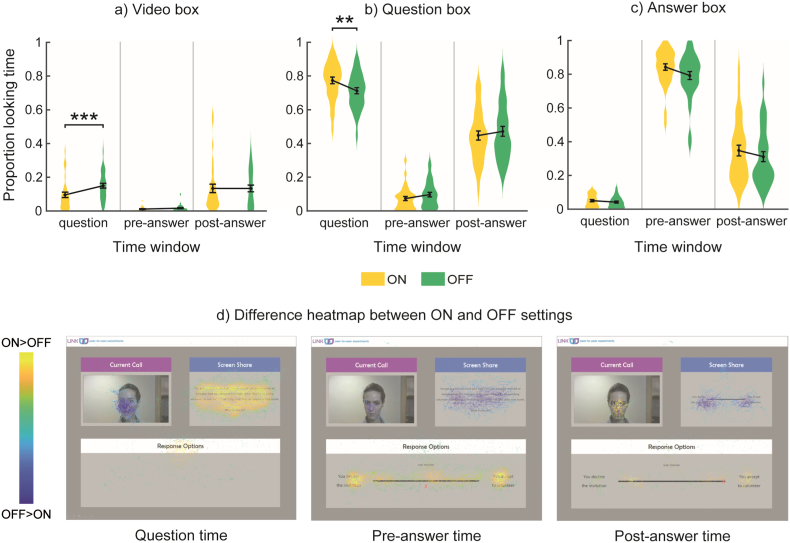
Story task. Proportion of looking time for each box, time window and setting: mean (●), SE (error bars), and frequency of values (width of distribution). a) Video box. b) Question box. c) Answer box. d) Heatmaps showing difference in proportion of looking time between ON and OFF settings for each box and time window. Asterisks signify difference between ON and OFF setting at *p* < .05 (*), *p* < .01 (**) and *p* < .001 (***).

For looking time to the Video box, there was a main effect of Time window, *F*(2,62) = 38.5, *p* < .001, n_p_^2^ = 0.554, and a tendency for an interaction effect between Setting and Time window, *F*(2,62) = 3.6, *p* = .054, n_p_^2^ = 0.104. Participants looked more to the Video box during the question and post-answer phases, especially in the OFF setting (Fig. 5a,d).

For looking time to the Question box, there was a main effect of Time window, *F*(2,62) = 437.1, *p* < .001, n_p_^2^ = 0.934, and an interaction effect between Setting and Time window *F*(2,62) = 5.81, *p* < .01, n_p_^2^ = 0.158. Participants looked more to the Question box in the question and post-answer phases, especially in the ON setting (Fig. 5b,d).

For looking time to the Answer box, there was a main effect of Setting, *F*(1,31) = 5.17, *p* < .05, n_p_^2^ = 0.143, and a main effect of Time window, *F*(2,62) = 710.1, *p* < .001, n_p_^2^ = 0.958, but no interaction effect between these two factors. Participants looked more to the Answer box in the pre-answer phase and in the ON setting (Fig. 5c,d).

Overall, these results are consistent with gaze shifting between the different windows as the task progresses, with less gaze towards the Video box and more towards the Question or Answer boxes in the ON setting, when participants believe the confederate can see them.

### 3.4 Eye gaze: offer task

For eye gaze, we fitted a two-way repeated measures ANOVA for each box (Video, Question, Answer), with Setting (ON and OFF) and Time window (question, pre-answer, post-answer) as within-subject factors. See Table 3 for descriptives (mean and SD) on the proportion of looking time to each box and time window. Full results are reported in Supplementary Materials (Table S6.2) and significant main effects and interactions are described below.

**Table 3:** Descriptives for the proportion of looking time to each box (Offer task).

Setting	Time window	Video box	Question box	Answer box
ON	question	*M = 0*.117 *SD = 0*.152	*M = 0*.730 *SD = 0*.150	*M = 0*.059 *SD = 0*.043
	pre-answer	*M = 0*.015 *SD = 0*.021	*M = 0*.217 *SD = 0*.127	*M = 0*.522 *SD = 0*.176
	post-answer	*M = 0*.165 *SD = 0*.155	*M = 0*.513 *SD = 0*.209	*M = 0*.136 *SD = 0*.108
OFF	question	*M = 0*.158 *SD = 0*.150	*M = 0*.652 *SD = 0*.160	*M = 0*.057 *SD = 0*.043
	pre-answer	*M = 0*.027 *SD = 0*.037	*M = 0*.209 *SD = 0*.124	*M = 0*.544 *SD = 0*.206
	post-answer	*M = 0*.247 *SD = 0*.153	*M = 0*.356 *SD = 0*.177	*M = 0*.147 *SD = 0*.092

**Fig. 6:**
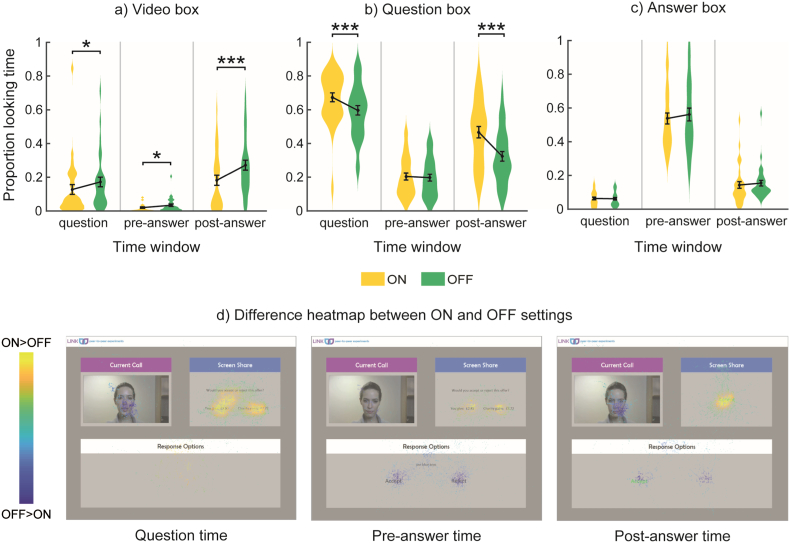
Offer task. Proportion of looking time for each box, time window and setting: mean (●), SE (error bars), and frequency of values (width of distribution). a) Video box. b) Question box. c) Answer box. d) Heatmaps showing difference in proportion of looking time between ON and OFF settings for each box and time window. Asterisks signify difference between ON and OFF setting at *p* < .05 (*), *p* < .01 (**) and *p* < .001 (***).

For the Video box, there was a main effect of Setting, *F*(1,31) = 13.5, *p* < .01, n_p_^2^ = 0.303, so that participants tended to look more to the Video box under the OFF setting compared to the ON setting. There was also main effect of Time window, *F*(2,62) = 37.0, *p* < .001, n_p_^2^ = 0.544, and an interaction effect between Setting and Time window *F*(2,62) = 8.0, *p* < .01, n_p_^2^ = 0.205: participants looked more to the Video box during the question and post-answer phases, especially in the OFF setting (Fig. 6a,d).

For the Question box, there was a main effect of Setting, *F*(1,31) = 23.5, *p* < .001, n_p_^2^ = 0.431: participants looked more to the Question box under the ON setting compared to the OFF setting. There was also a main effect of Time window, *F*(2,62) = 122.0, *p* < .001, n_p_^2^ = 0.797, and an interaction effect between Setting and Time window, *F*(2,62) = 21.3, *p* < .001, n_p_^2^ = 0.408: participants looked more to the Question box during the question and post-answer phases, especially in the ON setting (Fig. 6b,d).

For the Answer box, there was a main effect of Time window, *F*(2,62) = 210.7, *p* < .001, n_p_^2^ = 0.872, but no main effect of Setting or interaction: participants looked more to the Answer box in the pre-answer phase (Fig. 6c,d). These results are consistent with the Story task: gaze moves around the screen according to task demands, and participants look less to the confederate in the ON setting compared to the OFF setting.

Overall, these results are consistent with those obtained in the Story task: gaze moves around the screen according to the task demands, and participants look less to the video-feed in the ON setting compared to the OFF setting.

### 3.5 Relationship between prosocial behaviour and eye gaze

The data above shows that participants changed both their gaze behaviour and their prosocial choices according to whether they were being watched or not. Thus, it is useful to know if these two measures of social behaviour are related to each other on a trial-by-trial basis.

First, we tested if choices are related to previous gaze behaviour (during the question phase), that is, are people more prosocial when they look more to the video-feed? For this, we fitted a mixed ANOVA for each task, with Setting and Gaze (% looking time to Video box during question phase) as independent variables, Participant as random factor, and Choice as dependent variable. For the Story task, results showed that there was no main effect of Setting or Gaze, nor an interaction effect of Setting X Gaze, on prosocial choices (see Table 4a). For the Offer task, there was no strong evidence for a main effect of Setting or Gaze (see Table 5a).

**Table 4:** Relationship between prosocial behaviour and eye gaze (Story task).

a) Does gaze before the choice predicts choices?		b) Do choices predict gaze after the choice?	
Setting	*Beta* = −0.013 *t*(293.8) = −0.243 *p* > .05	Setting	*Beta* = −0.022 *t*(288.2) = −0.641 *p* > .05
GazeBefore	*Beta* = −0.246 *t*(303.8) = −0.973 *p* > .05	Choice	*Beta* = −0.106 *t*(303.8) = −2.68 *p* < .01^⁎⁎^
Setting X GazeBefore	*Beta* = −0.159 *t*(302.9) = −0.506 *p* > .05	Setting X Choice	*Beta* = 0.031 *t*(289.6) = 0.586 *p* > .05
Participant	*Beta* = 0.022 *Z* = 2.65 *p* < .01^⁎⁎^	Participant	*Beta* = 0.009 *Z* = 3.04 *p* < .01^⁎⁎^

**Table 5:** Relationship between prosocial behaviour and eye gaze (Offer task).

a) Does gaze before the choice predicts choices?		b) Do choices predict gaze after the choice?	
Setting	*Beta* = −0.049 *t*(1,506.9) = −1.79 *p* = .074^+^	Setting	*Beta* = 0.084 *t*(30.5) = 3.40 *p* < .01^⁎⁎^
GazeBefore	*Beta* = −0.156 *t*(1,450.9) = −1.67 *p* = .09^+^	Choice	*Beta* = −0.019 *t*(33.1) = −0.691 *p* > .05
Setting X GazeBefore	*Beta* = 0.139 *t*(1,519.5) = 1.26 *p* > .05	Setting X Choice	*Beta* = 0.031 *t*(289.6) = 0.586 *p* > .05
Participant	*Beta* = 0.033 *Z* = 27.4 *p* < .001^⁎⁎⁎^	Participant	*Beta* = 0.009 *Z* = 3.04 *p* < .01^⁎⁎^

Second, we tested if choices are related to gaze behaviour in the post-answer phase: do participants look to the confederate to see if she evaluates their choice? For this, we fitted a mixed ANOVA for each task, with Setting and Choice as independent variables, Participant as random factor, and Gaze (% looking time to Video box during post-answer phase) as dependent variable. For the Story task, the proportion of looking time to the Video box after giving an answer was negatively predicted by the prosociality of that answer, *Beta* = −0.106, *t* = −2.68, *p* < .01 (see Table 4b), although there was no interaction between Setting and Choice. This means that a decrease in the prosociality of the choices was associated with an increase in the proportion of looking time to the Video box during the post-answer time window, regardless of belief. For the Offer task we found a main effect of Setting, *Beta* = −0.084, *t* = 3.40, *p* < .01 (see Table 5b): participants looked more to the Video box under the OFF setting, regardless of the type of choice.

## 4 Discussion

The present study aimed to examine audience effects on prosocial and gaze behaviour, and test whether they can be explained in terms of reputation mechanisms. More specifically, we show the following. First, we show that prosocial behaviour (both disclosure of prosocial tendencies and monetary donations) somewhat increases when it is possible to signal a good reputation to an observer. We also find that the increase of prosocial behaviour when disclosing prosocial tendencies positively correlates with social anxiety traits. Second, we extend findings from non-communicative studies by showing that gaze signalling also conforms to a social norm of avoiding staring in communicative situations. Finally, we find that participants look longer towards the confederate after making a non-prosocial choice, but this is true for both the live and pre-recorded interactions. These findings also show that the deceptive video-conference paradigm is an efficient experimental setting to test audience effects. The implications of these findings for social cognitive research are discussed below.

### 4.1 Reputation management and being watched

Using our novel deceptive video-conference paradigm we find marginal evidence that, both in the Story and Offer tasks, participants are more likely to act for the benefit of other people (i.e. they choose more prosocially) when they believe they are being watched than when they do not hold this belief. This corroborates previous studies showing that people increase their prosocial behaviour when being watched (Cage et al., 2013; Emler, 1990; Filiz-Ozbay & Ozbay, 2014; Izuma et al., 2010, Izuma et al., 2009; Izuma et al., 2011; Satow, 1975; Tennie et al., 2010). Because control and test conditions in our paradigm are tightly matched (we use the same stimuli across both ON and OFF settings), they differ only in the belief in being watched. Thus, these findings indicate that this change in behaviour may be driven by the need to signal good reputation in front of an observer (Bradley et al., 2018; Smith & Bird, 2000), rather than by the mere presence of another person. A key element in reputation management is that individuals seek to be viewed positively by others (Cage, 2015; Izuma, 2012), and achieving this is processed as a social reward (e.g. Izuma et al., 2009; Izuma et al., 2010). In the context of our tasks, the social reward associated with making prosocial choices in front of others likely exceeds the individual temporal or monetary benefits associated with non-prosocial choices.

Although audience effects on prosocial behaviour are marginal in both tasks, we find that they are somewhat stronger in the Story task than in the Offer task. This suggests that changes in prosocial behaviour in lab-based studies happen beyond decisions made in economic games (Cage et al., 2013; Filiz-Ozbay & Ozbay, 2014; Izuma et al., 2011), that is, even when decisions apply to daily life situations. Given that economic games may have poor external validity (Galizzi & Navarro-Martinez, 2017; Winking & Mizer, 2013), it would be interesting to see how our findings generalise to real world contexts. This might be a promising (and challenging) avenue for future research on audience effects.

There are several possible reasons why, compared to previous studies (Cage et al., 2013; Izuma et al., 2011), we find only a tendency for an audience effect in the Offer task. On the one hand, in previous studies participants were given an endowment of around £40 (payment for attending a full testing day), but in our experiment participants were given an endowment of only £4: this amount might be too low to make participants feel they are losing money if they decide to donate it. On the other hand, in previous studies participants would have a 50–90 min break between the two sessions/settings, whereas in our study there was no break. This could explain the trend towards an effect of the order in which the settings appeared: doing the task first under the ON setting seemed to have a carryover effect of being watched on prosocial behaviour in the OFF setting. Finally, our study is somewhat underpowered to detect effects of being watched on prosocial behaviour (see Limitations section below). One way to explore the effectiveness of our method further is to compare the behaviour of the 9 participants who did not believe our manipulation to the 32 who did, and we report this comparison in detail in the Supplementary Materials (S7). Briefly, the analysis suggests that believing the manipulation is critical to obtaining our results.

Interestingly, we find that higher social anxiety traits correlate with greater increase of prosocial behaviour in the Story task when being observed. These findings are in line with previous evidence suggesting that people with social anxiety traits might be more susceptible to audience effects and reputation management. For instance, negative personality traits (e.g. low self-esteem, neuroticism or introversion, which are associated with social anxiety) are strong predictors of how social presence will affect individual performance (Uziel, 2007). Moreover, it has been shown that the need for social approval has a positive effect on the amount of money participants donate, particularly when donations are made in front of an observer (Satow, 1975). Our exploratory analysis corroborates these studies by showing that people with social anxiety traits, who have increased concerns to gain social approval (Cremers & Roelofs, 2016; Morrison & Heimberg, 2013), are more likely to change their behaviour (to signal good reputation) when other people are observing. However, this correlation is not found for the Offer task. A reason for this could be that economic games, such as the Dictator game used in the Offer task, have poor external validity (Galizzi & Navarro-Martinez, 2017; Winking & Mizer, 2013), so changes in this measure may not be sensitive to real-life behaviours rated in the social anxiety questionnaire.

### 4.2 Gaze behaviour and being watched

Gaze behaviour was recorded throughout the Story and Offer tasks to determine how people use gaze to gain and signal social information during a communicative interaction. Overall, both tasks show the same pattern of results. As expected, participants looked more at the Video and Question boxes when the question was asked, and more at the Answer box before giving an answer. An interesting pattern emerged with regard to the comparison between ON and OFF settings. During the question phase, participants spent less time looking at the Video box in the ON setting than in the OFF setting, while the opposite was found for the Question box. The same applied during the post-answer phase, although this was only true for the Offer task.

According to the dual function model of eye gaze (Gobel et al., 2015; Risko et al., 2016), these findings indicate that, when participants believe they are being watched, they use their gaze to signal to the other person and not just to acquire information. Averted gaze in live social interactions has been associated with preference for no interaction (Foulsham et al., 2011) and conformity with social norms (e.g. it is not polite to stare at someone; Gobel et al., 2015; Gobel et al., 2017; Laidlaw et al., 2011). Thus, it seems that in a communicative situation, gaze signalling also conforms to the social norm of avoiding staring, despite the closer social link between the participant and confederate. In line with this, the analysis with the group of excluded participants suggests that this pattern of results is specific to the group of participants who believe the manipulation (see S7). However, this finding contrasts with a recent study by Mansour and Kuhn (2019), where they find that participants in a communicative situation direct more gaze to the eyes of the confederate in a live video-call than in a pre-recorded video-call. A critical difference is that in their paradigm the confederate was talking about herself for around 2.5 min in a rather relaxed context, whereas in our tasks the confederate asked a short question of around 10 s (Story task) or 3 s (Offer task) in a more rigid context. As Mansour & Kuhn suggest, it could be that different social norms of eye gaze apply to different communicative situations: looking to the confederate to show interest is likely to be the norm when she is sharing personal information, whereas civil inattention may be the norm for more structured forms of interaction.

To further understand the meaning of these gaze patterns it is critical to consider the function of gaze as a social, but also interactive signal. The claim that gaze patterns change to conform to social norms provides a useful description of behaviour (Gobel et al., 2015; Gobel et al., 2017; Laidlaw et al., 2011), but this is not the same as having a detailed cognitive model of the control of social gaze. Such a model should integrate temporal and spatial aspects of gaze across different contexts to give a sensible account of eye gaze in real life, but also a more accurate interpretation of previous studies using photos and videos. In the following, we show how analysing the relationship between eye gaze and other behaviours (prosocial choices) can help identifying social cognitive mechanisms that modulate eye gaze in live interactions.

### 4.3 Relationship between prosocial and gaze behaviour

To our knowledge, our study is the first one to simultaneously measure prosocial behaviour and eye gaze in a conversation context: this creates a suitable communicative environment to examine the relationship between prosocial choices and gaze behaviour, and how they are modulated by the belief in being watched. In our design, we distinguish between three time windows (question, pre-answer and post-answer) locked to a key event in the interaction: the participant making a choice. We consider two different hypotheses.

The social attention hypothesis suggests that gaze behaviour at the start of the trial will predict later choices. For instance, it has been shown that mutual gaze increases prosocial behaviour of participants (see Bull & Gibson-Robinson, 1981 for an example). In both the Story and Offer task, there was no evidence to support this: looks at the start of the trial did not relate to subsequent choices in either setting. This suggests that the amount of attention directed to the confederate does not impact on prosocial decision-making.

The reputation management hypothesis suggests that prosocial choices will predict gaze behaviour after the choice in the ON setting, because participants will look at the confederate to seek information about how they are evaluated (e.g. check if she approves or disapproves their choices) (Efran, 1968; Efran & Broughton, 1966; Kleinke, 1986). For the Story task, we find that participants looked more to the confederate after making a non-prosocial choice than a prosocial choice, but this is true for both ON and OFF settings. Although this is not entirely consistent with the reputation management hypothesis (the effect was found in both ON and OFF settings; discussed below), it suggests that participants were generally worried about what the confederate would think of them when they made a non-prosocial choice: by gazing to the confederate, participants could monitor whether she disapproved their choice, and gave them the chance to re-engage with her again. In line with this, Nasiopoulos, Risko, and Kingstone (2015) have recently suggested that participants' gaze may weigh the potential gain of attending to a specific location with the cost of revealing their attentional state. In the context of our task, both attending to what the confederate thinks and revealing that ‘I want to re-engage with her’ are strongly beneficial to restore reputation after making a non-prosocial choice, and this might result in more looking to the confederate. Moreover, we did not find this relationship in the group of excluded participants (see S7), which indicates that the feeling that the confederate can evaluate their choices fades away once the manipulation is uncovered.

There are two main limitations to this result. First, we could not replicate this finding in the Offer task. It could be that participants care more about reactions to the choices in the Story task because they are more meaningful to them (i.e. they depict real-life situations). However, it is necessary that future studies test whether this relationship is also true for other types of prosocial choices. Second, this relationship was not modulated by the belief in being watched: participants behaved equally in ON and OFF settings. It is not yet clear if this is because of too much social gaze in the OFF setting (OFF is like ON) or too little social gaze in the ON setting (ON is like OFF). The former could arise if there is a default response of acting in a social fashion whenever we are in front of a social stimulus, and if top-down knowledge that ‘this is not a real person’ is not enough to inhibit the natural social behaviour. Similar effects are seen when a person gestures even when talking on the telephone, despite knowing that the other cannot see them. Alternatively, it could be that our video-conference condition is not a perfect match for real life, because it is a computer-mediated interface without true eye contact. Thus, participants might not engage in social signalling as fully as they would in real life. Further studies comparing face-to-face interactions with video-conferencing and video watching conditions will help distinguish between these possibilities. Overall, our findings suggest that the belief in being watched has different degree of modulation over prosocial choices and eye gaze, but that it may not be sufficient to fully modulate complex social behaviour like the relationship between prosocial choices and gaze.

### 4.4 Limitations

Although these are promising findings for cognitive research on audience effects, the design of this study also has some general limitations. First, there is not enough evidence for a strong effect of being watched on prosocial behaviour. Post-hoc power analyses with G*Power (Faul, Erdfelder, Buchner, & Lang, 2009; Faul, Erdfelder, Lang, & Buchner, 2007) showed that the study is underpowered to detect effects of being watched on prosocial behaviour in both tasks (power ≈ 0.5), but is well powered to detect effects of being watched on gaze (power ≈ 0.9). This could be due to low number of behavioural trials (5 in the Story task, and 24 in the Offer task), in contrast with the large number of data-points collected for eye-tracking. Keeping the number of behavioural trials low was essential to keep the study short and increase ecological validity (i.e. with too many repetitions it would be easy to detect that the confederate was always pre-recorded). Future studies with bigger sample sizes would increase power and yield enough evidence to reliably find (or not) an effect of Setting on prosocial behaviour in both tasks. However, we do not think that finding strong effects on prosocial behaviour is fundamental for the rest of the study (i.e. eye gaze results). The fact that eye gaze (a quick and spontaneous behaviour) is strongly modulated by Setting, but making prosocial choices (a strategic decision-making process) shows weaker modulation, suggests that different forms of reputation management have different sensitivity to the belief in being watched, at least when using our deceptive video-conference paradigm.

Second, we find that evidence for audience effects on prosocial behaviour is stronger in the Story task than in the Offer task, also when testing the relationship with social anxiety traits. Although this could be due to the different nature of the questions asked in each task (disclosure of prosocial tendencies in real-life situations, or monetary decisions in an economic game), it is important to consider that participants always completed the tasks in the same order: Story task followed by Offer task. Thus, it could be that after completing the Story task participants feel more relaxed towards the confederate monitoring their choices, and consequently do not change their prosocial behaviour in the Offer task. Counterbalancing the order of the tasks would clarify whether some of these effects are also found when using more artificial tasks like economic games.

One last concern is the gaze metric we use, proportion of total looking time. It has been suggested that this type of metric can challenge internal validity, because it involves inappropriate aggregation of gaze data (Orquin & Holmqvist, 2018). For instance, when we find that participants look more to the Video box in the OFF setting, it could be that there are many short fixations, or that fixations are longer. Thus, using more precise measures such as number of fixations and fixation duration can be more informative to accurately interpret gaze data.

### 4.5 Implications and future research

The present findings have important implications for social neuroscience research. We show that our deceptive video-conference paradigm is effective in promoting cognitive processes triggered by the belief in being watched (e.g. reputation management, signalling function of gaze), while combining high ecological validity and experimental control. Interestingly, we also find that under the belief in being watched the confederate is perceived as more likeable and natural, and tends to be perceived as more reciprocal: being embedded in a true interaction and able to communicate with each other modulates how we behave in front of others, but also has positive consequences on how we perceive our interactive partners. This is supported by the analyses with participants who do not believe the deceptive manipulation, since they perceive both confederates as equally likeable, natural and reciprocal. In light of these results and following advocates for a second-person neuroscience (Risko et al., 2016; Schilbach et al., 2013), we encourage researchers to take a more ecologically valid approach when implementing studies on social cognition, either by having a real interaction or by using alternative approaches, such as this deceptive video-conference paradigm.

We also provide novel evidence of how relationships between gaze and other events in the interaction can potentially help identify social cognitive processes that modulate gaze behaviour. Here, the relationship between prosocial choices and subsequent eye gaze suggests that reputation management engages a strategic use of gaze to maintain reputation: the less prosocial choices are, the more participants look to the confederate to monitor how they are evaluated. This finding highlights the importance of the relationship between gaze and other events in the interaction (such as whether ‘I am behaving in a prosocial way or not’) in understanding gaze behaviour in live communicative contexts. However, future studies should investigate whether this is a spontaneous gaze response that is normally inhibited in non-live settings, and whether face-to-face interactions (where both partners directly see each other) boost the effects on this relationship. Overall, cognitive models that explain changes of eye gaze in real life need to incorporate its dynamic and interactive aspects: this will be key to understand gaze behaviour in real life, but also to carefully re-interpret previous studies using photos and videos.

### 4.6 Conclusion

The present study aimed to advance current knowledge of how prosocial and gaze behaviour acquire a signalling function when being watched, and whether this can be explained by reputation management processes. By using our novel deceptive video-conference manipulation and a communicative context, we show that under the belief in being watched participants tend to increase prosocial decisions, and that this increase correlates with social anxiety traits. We also find that when being watched participants modulate their gaze according to social norms. This extends previous findings in non-communicative situations and indicates that participants change their prosocial and gaze behaviour to signal good reputation to others. To our knowledge, we also show for the first time that prosocial choices influence subsequent gaze patterns of participants. Overall, these results suggest that reputation mechanisms modulate both prosocial and gaze behaviour, and indicate that gaze should be considered as an interactive signal. They also highlight the need to build up a cognitive model of gaze dynamics in live interactions.

## Compliance with ethical standards

The authors declare no conflicts of interest. All procedures were approved by the UCL Research Ethics Committee and were in accordance with the Declaration of Helsinki and APA ethical standards.
